# Dimethyloxaloylglycine-stimulated human bone marrow mesenchymal stem cell-derived exosomes enhance bone regeneration through angiogenesis by targeting the AKT/mTOR pathway

**DOI:** 10.1186/s13287-019-1410-y

**Published:** 2019-11-20

**Authors:** Bo Liang, Jia-Ming Liang, Jia-Ning Ding, Jia Xu, Jian-Guang Xu, Yi-Min Chai

**Affiliations:** Department of Orthopedic Surgery, Shanghai Jiao Tong University Affiliated Sixth People’s Hospital, Yishan Rd 600, Shanghai, 200233 People’s Republic of China

**Keywords:** Exosome, Mesenchymal stem cell, Bone regeneration, Angiogenesis, Dimethyloxaloylglycine, Tissue engineering

## Abstract

**Background:**

Mesenchymal stem cell (MSC)-derived exosomes have been recognized as new candidate agents for treating critical-sized bone defects; they promote angiogenesis and may be an alternative to cell therapy. In this study, we evaluated whether exosomes derived from bone marrow-derived MSCs (BMSCs) preconditioned with a low dose of dimethyloxaloylglycine (DMOG), DMOG-MSC-Exos, exert superior proangiogenic activity in bone regeneration and the underlying mechanisms involved.

**Methods:**

To investigate the effects of these exosomes, scratch wound healing, cell proliferation, and tube formation assays were performed in human umbilical vein endothelial cells (HUVECs). To test the effects in vivo, a critical-sized calvarial defect rat model was established. Eight weeks after the procedure, histological/histomorphometrical analysis was performed to measure bone regeneration, and micro-computerized tomography was used to measure bone regeneration and neovascularization.

**Results:**

DMOG-MSC-Exos activated the AKT/mTOR pathway to stimulate angiogenesis in HUVECs. This contributed to bone regeneration and angiogenesis in the critical-sized calvarial defect rat model in vivo.

**Conclusions:**

Low doses of DMOG trigger exosomes to exert enhanced proangiogenic activity in cell-free therapeutic applications.

## Background

Critical-sized bone defects caused by severe trauma, tumor resection, and congenital defects cannot be repaired without orthopedic intervention [1]. Autologous and allogeneic bone grafting are commonly used to promote the healing of bone defects but the limited supply, high costs, and rejection risks prevent their widespread use [2]. Thus, bone tissue engineering has emerged as a therapeutic strategy. This approach involves the use of various cells and biological factors in combination with bone substitutes to improve their osteogenic and angiogenic activities. Angiogenesis is a prerequisite to bone healing, and restoration of the blood supply to bone defects provides a rich source of growth factors and nutrients [3]. Therefore, how to promote angiogenesis in tissue-engineered bone is a critical area of research.

Mesenchymal stem cells (MSCs) are multipotent cells with differentiation abilities, immunomodulatory effects, and homing properties and may significantly augment the regenerative capacity of many tissues [4]. MSCs play an important role in neovascularization, such as myocardial infarction [5], hind limb ischemia [6], wound healing [7], and bone repair. MSCs secrete angiogenic growth factors that promote local revascularization. Extracellular vesicles (EVs) comprise diverse types of membrane vesicles released from cells into the extracellular environment and participate in intercellular communication [8]. There are three main subtypes of EVs, exosomes, microvesicles (MVs), and apoptotic bodies, which show different characteristics based on size, function, content, biogenesis, and release pathways [9, 10]. As a subtype of EVs, exosomes can be secreted by almost all kinds of cells, are formed by an endosomal route, are enclosed within a single outer membrane, and normally have 30–150 nm in diameter [8]. Exosomes were previously considered an instance of cellular dumping, or a functional way for cells to dispose of unneeded material. However, exosomes are now known to be involved in the paracrine effects of cells, containing many active molecules of its source cells to fulfill the transportation and exchange of signals among cells [11]. MSCs produce massive amounts of exosomes, and many regenerative properties previously credited to stem cells may actually be attributed to secreted exosomes [12]. Thus, in addition to MSC-secreted cytokines, MSC-derived extracellular vesicles, including exosomes [13] and microvesicles [14], also promote angiogenesis. Though the value of clinical applications of exosomes has been noticed many years ago, a key obstacle for its clinical use is the lack of efficiency, stabilization, and standardization in its isolation methods [15]. With the establishment and development of many isolation methods in the past decade, especially the ultracentrifugation method that is now recognized as the gold standard, the isolation of exosomes is now very efficient and has low cost, facilitating its comprehensive study and clinical use in the future [16, 17].

Preconditioning of MSC culture conditions via hypoxia, pharmacological agents, chemical agents, trophic factors, cytokines, and physical factors is a key strategy for improving MSC function in vitro and in vivo [18–23].. For example, transplantation of MSCs under hypoxia conditions significantly potentiates the survival and anti-apoptosis ability of MSCs. Hypoxic preconditioning not only increases MSC-induced angiogenesis and neuroprotection but also improves the proliferation and migration of MSCs, thereby improving the efficiency of MSC transplantation [24]. Exosomes from hypoxic cardiac progenitor cells, which improve cardiac function and reduce fibrosis after ischemia–reperfusion injury, have been found to contain 11 miRNAs with upregulated expression compared to their levels in exosomes secreted by normoxic cardiac progenitor cells [25]. Moreover, hypoxia treatment enhanced microvesicle production by umbilical cord-derived MSCs, promoting in vitro capillary-like structure formation and improved blood flow recovery in a rat hindlimb ischemia model [14]. Exosomes derived from hypoxia-preconditioned MSCs promoted neovascularization and graft survival in fat grafting [26]. Compared to MSCs themselves, use of their exosomes may improve efficiency in some areas, as they can be isolated and concentrated to amplify their source cell’s active molecules and avoid the physical constraints of intercellular contact. Furthermore, the cell-free attribute of exosome application could overcome many cell-related problems such as cell fate control [27, 28].

It is well known that these MSC enhancements caused by hypoxia are mainly mediated by the hypoxia-inducible factor (HIF) complex. Under hypoxic conditions, its HIF-1α subunit translocates to the nucleus, where HIF complexes with other components to initiate transcription of HIF target genes [29]. DMOG is a small angiogenic molecule that inhibits prolyl hydroxylase to regulate the stability of HIF-1α, mimicking hypoxia in cells under normal oxygen levels [30–32]. Our previous studies showed that pretreating bone marrow-derived MSCs (BMSCs) with DMOG activates the expression of hypoxia-inducible factor-1α, thereby improving the angiogenesis of tissue-engineered bone and its bone healing capacity [33, 34]. However, it remained unclear whether exosomes released from MSCs pretreated with DMOG-enhanced angiogenic activity in vitro or in vivo.

Therefore, this study was conducted to investigate whether exosomes from BMSCs preconditioned with a low dose of DMOG have superior proangiogenic properties and promote angiogenesis and bone regeneration in a critical-sized calvarial defect rat model. We also investigated the possible underlying mechanisms.

## Methods

### Cell culture

Human BMSCs (ScienCell, Carlsbad, CA, USA) were cultured in minimum essential medium, alpha medium (Corning, Inc., Corning, NY, USA), and 10% fetal bovine serum (Gibco, Grand Island, NY, USA). Human umbilical vein endothelial cells (HUVECs; ScienCell) were cultured in endothelial cell medium (ScienCell) containing 5% fetal bovine serum (ScienCell) and 1% endothelial cell growth supplement (ScienCell). For DMOG stimulation, BMSCs were exposed to 1000 μM DMOG (Sigma-Aldrich, St. Louis, MO, USA) for 48 h under culture conditions.

### Exosome purification

After 48 h of culture, the BMSC medium was harvested and centrifuged at 500×*g* for 10 min to remove the cells. The supernatant was then centrifuged at 12,000×*g* for 20 min to remove apoptotic bodies and cell debris, followed by filtration through a 0.22-μm filter to remove molecules larger than 200 nm. Exosomes were then pelleted by ultracentrifugation at 110,000×*g* for 70 min (Optima™ XPN, 45Ti, Beckman, Brea, CA, USA). The resulting pellet was further purified by re-suspension in phosphate-buffered saline (PBS) and ultracentrifuged at 110,000×*g* for another 70 min to remove contaminating protein. Exosome pellets were resuspended in PBS and stored at − 80 °C.

### Exosome characterization and internalization

Transmission electron microscopy (TEM; HT7700, Hitachi, Tokyo, Japan) was performed for morphological analysis of isolated exosomes. The absolute size distribution of exosomes was determined using the qNano platform (iZON® Science, Christchurch, New Zealand). Western blotting of proteins (CD9, CD63, TSG101, and GM130) in exosomes was conducted as described previously [23] with the following primary antibodies: CD9 (1:1000; rabbit IgG, Proteintech, Rosemont, IL, USA), CD63 (1:1000; rabbit IgG, Proteintech), TSG101 (1:1000; rabbit IgG, Proteintech), and GM130 (1:500; rabbit IgG, Abcam, Cambridge, UK). Exosomes were then labeled with green fluorescent dye (DIO; Life Technologies, Carlsbad, CA, USA), and excess dye was removed by ultracentrifugation at 110,000×*g* for 70 min at 4 °C. Exosome pellets were washed three times and resuspended in PBS. HUVECs were incubated with DIO-labeled exosomes for 8 h, and cell nuclei were stained with 4,6-diamidino-2-phenylindole (DAPI; Southern Biotech, Birmingham, AL, USA); uptake was observed by fluorescence microscopy.

### Western blotting

Proteins were isolated using RIPA lysis solution (Santa Cruz Biotechnology, Dallas, TX, USA). Protein concentrations were determined using the Pierce BCA Protein Assay Kit (Thermo Fisher Scientific, Waltham, MA, USA). Equal amounts of protein were separated by sodium dodecyl sulfate-polyacrylamide gel electrophoresis and transferred to polyvinylidene fluoride membranes. The membranes were blocked with 5% milk in Tris-buffered saline containing 0.1% Tween 20 and incubated with primary antibodies as follows: PTEN (1:1000, Abcam), AKT (1:1000; Cell Signaling Technologies, Danvers, MA, USA), p-AKT (1:1000; Cell Signaling Technologies), mTOR (1:1000; Abcam), p-mTOR (1:1000; Abcam), and actin (1:10,000; Thermo Fisher Scientific). Membranes were incubated with appropriate horseradish peroxidase-conjugated secondary antibody against rabbit (1:1000) or mouse (1:4000) IgG (Jackson Laboratories, Bar Harbor, ME, USA). Actin was used as a loading control.

### Quantitative real-time PCR

Total RNA was extracted using TRIzol reagent, and cDNA was synthesized using 4× Reverse Transcription Master Mix (EZBioscience, Roseville, MN, USA). Gene expression was quantified by quantitative real-time PCR (qRT-PCR) using FastStart Universal SYBR Green Master (Roche, Basel, Switzerland).

### Cell proliferation assay

HUVECs were seeded at 2 × 10^3^ cells/well into 96-well plates, and exosomes derived from MSCs (MSC-Exos) and DMOG-stimulated MSCs (DMOG-MSC-Exos) (50 mg/mL) or an equivalent volume of exosome diluent (PBS) was added to the culture medium for 4 days. Cell Counting Kit-8 (CCK8; Dojindo Laboratories, Kumamoto, Japan) was used for cell proliferation assays. The optical density (OD) was measured at 450 nm with a microplate reader.

### Scratch wound healing assay

HUVECs were seeded at 2 × 10^5^ cells/well into six-well plates. At 90% confluence, scratch wounds were made across each well using a sterile plastic 100-μL micropipette tip. After washing each well twice with PBS, basal DMEM-containing exosomes at a final concentration of 50 mg/mL was added. Images of each scratch were taken from three fields of view (× 100 magnification) at 0, 6, and 12 h. ImageJ software (NIH, Bethesda, MD, USA) was used to evaluate migration by measuring the residual fractional wound area.

### Tube formation assay

In vitro capillary network formation was evaluated by a tube formation assay on Matrigel (BD Biosciences, Franklin Lakes, NJ, USA). HUVECs were seeded at 2 × 10^4^ cells per well onto a Matrigel-coated 24-well plate and cultured in the presence of exosomes at the same concentrations as those indicated in the cell proliferation assay (50 mg/mL) for 16 h at 37 °C. Each concentration was evaluated in triplicate. After incubation for 16 h, tube formation was examined by microscopy, and total tube length was quantified by analyzing three randomly selected fields per well with ImageJ software.

### Animal experiments

All animal procedures were approved by the Animal Research Committee of the Sixth People’s Hospital, Shanghai Jiao Tong University. Classical porous hydroxyapatite (HA) scaffolds (5-mm diameter, 2-mm depth) with an average pore size of 500 μm and 75% porosity were used as cell carriers for in vivo studies. Thirty rats were randomly divided into three groups to receive the following implants: (1) HA (*n* = 10), (2) HA with MSC-Exos (*n* = 10), and (3) HA with DMOG-MSC-Exos (*n* = 10). Adult male Sprague-Dawley rats weighing 250–300 g were anesthetized, and two 5-mm diameter critical-sized calvarial defects were created on each side of the cranium using a dental trephine. Scaffolds were then implanted into the defects, and 100 μg of exosomes in 200 μL PBS or 200 μL PBS alone (control) was injected into the implanted scaffolds. Eight weeks after surgery, five rats per group were sacrificed, and their craniums were harvested. The craniums were analyzed by micro-computed tomography (CT), followed by non-decalcified histological analysis (see below). To compare blood vessel formation in vivo, at 8 weeks after surgery, five rats per group were anesthetized for rib cage opening. The descending aorta was clamped, and an angiocatheter was used to penetrate the left ventricle. After the inferior vena cava was incised, heparinized saline was perfused until the venous effluent was free from blood. Next, 20 mL Microfil (Flow Tech) was perfused at 2 mL/min. Finally, the rats were maintained overnight at 4 °C to ensure polymerization of the contrast agent. To further evaluate the neovascularization effects in vivo, immunofluorescence analysis for CD31 was also performed.

### Sequential fluorescent labeling

At 2, 4, and 6 weeks after surgery, the rats were intraperitoneally injected with tetracycline (25 mg/kg body weight; Sigma), alizarin red (30 mg/kg body weight; Sigma), and calcein (20 mg/kg body weight; Sigma). Trichromatic sequential fluorescent labeling was then used to observe mineralized tissue.

### Micro-CT analysis

At 8 weeks after surgery, five rats per group were sacrificed. The craniums were fixed in 10% neutral-buffered formalin solution for at least 48 h. Undecalcified and decalcified samples perfused with Microfil were scanned using micro-CT (Skyscan, Bruker, Billerica, MA, USA) at resolutions of 18 and 9 μm, respectively. A threshold of 800 was defined as bone tissue. Three-dimensional grayscale images were generated using auxiliary software. Percentages of new bone volume relative to tissue volume (BV/TV) in the bone defects area were calculated, and local bone mineral densities (BMDs) were determined using the analysis software (CTan, Bruker, Billerica, MA, USA).

### Histological analysis

One part of each fixed cranium was dehydrated through a graded alcohol series and then embedded in polymethylmethacrylate. After hardening, sagittal sections of the central segment were cut into 150- to 200-μm-thick slices using a microtome (Leica, Wetzlar, Germany), glued onto a plastic support, and polished to a final thickness of approximately 40 μm. A confocal laser scanning microscope (Leica) was used to observe the fluorescent labeling of the sections using excitation/emission wavelengths for chelating fluorochromes of 405/580 nm (tetracycline, yellow), 543/617 nm (alizarin red, red), and 488/517 nm (calcein, green). The sections were then stained with van Gieson’s picrofuchsin to assess new bone formation (red and black indicated new bone and HA, respectively).

### Immunofluorescence analysis for CD31

Immunofluorescence analysis for CD31 (1:200, Abcam, UK) was also performed to evaluate the formation of new blood vessels in each group. Briefly, a set of sections was rehydrated, boiled in 0.01 M citrate buffer (pH 6.0) for 15 min, and then blocked with 5% goat serum PBS for 30 min at room temperature. Following blocking, sections were incubated with the primary antibody at 4 °C overnight. Subsequently, the sections were incubated with the secondary antibody (1:400, Abcam, UK) for 1 h. The sections were incubated with DAPI to stain the nucleus and observed with a fluorescence microscope (Leica, Germany). The formation of blood vessels was evaluated by the positive CD31 staining area and structures.

### Statistical analysis

Data are shown as the means ± standard deviations. Differences between groups were assessed by one-way analysis of variance using GraphPad Prism software (GraphPad, Inc., La Jolla, CA, USA); *P* values < 0.05 were considered to indicate statistically significant differences between groups.

## Results

### Exosome isolation, characterization, and internalization

The exosomal marker proteins CD9, CD63, GM130, and TSG101 were detected in exosomes derived from BMSC culture supernatants as expected, and no significant differences were observed on the expression of these markers between the MSC-Exos and DMOG-MSC-Exos groups (Additional file 1: Figure S1a). TEM analysis showed that exosomes were round, membrane-bound vesicles ranging from 30 to 100 nm in diameter, with no obvious differences in morphology and quantity between the MSC-Exos and DMOG-MSC-Exos groups (Additional file 1: Figure S1b). qNano analysis shows that the size of the exosomes mainly ranged from 80 to 182 nm, with a mean size of 130 nm, and the predicted proper concentration was 6.65 × 10^8^ particles/mL, with no distinguishing differences between the two exosome groups (Additional file 1: Figure S1c).

**Fig. 1:**
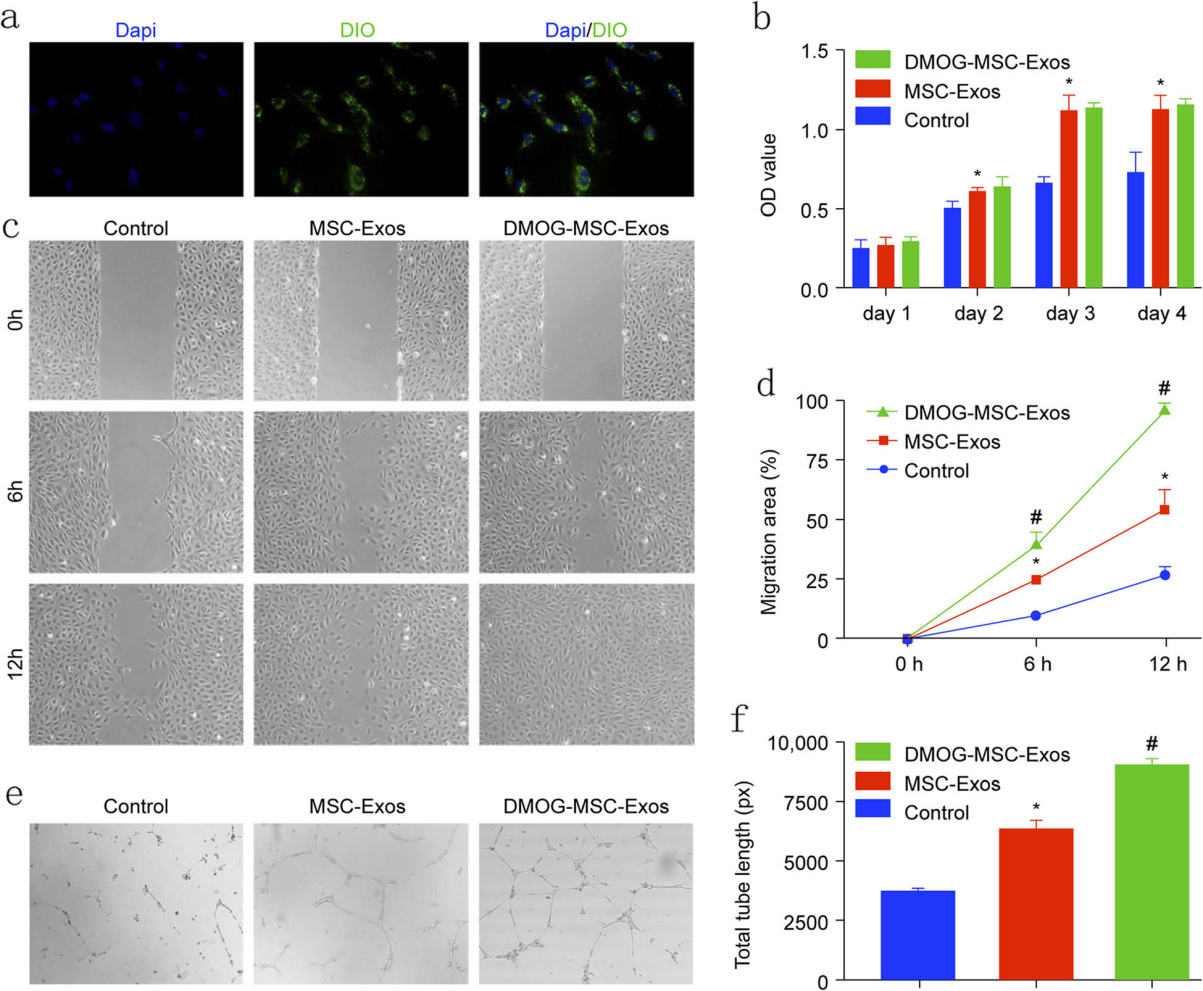
Internalization and proangiogenic effects of MSC-Exos in HUVECS. **a** Fluorescent microscopy showing internalization of DIO-labeled DMOG-MSC-Exos by HUVECS. Green-labeled exosomes are visible in the perinuclear region of HUVECs. **b** Proliferation of HUVECS treated with exosomes (50 μg/mL). **c** Wound healing assay of HUVECs treated with exosomes (50 μg/mL). **d** Quantitative analysis of wound closure. **e** Tube formation capacity of HUVECs treated with exosomes. **f** Quantitative analysis of total tube length (*P < 0.05, versus control; ^#^*P* < 0.05, versus MSC-Exos)

Exosome uptake by HUVECs was confirmed by fluorescence microscopy. After 8 h, over 90% of HUVECs were DAPI-positive, indicating that the DIO-labeled exosomes had been taken up and transferred into the cytoplasmic compartments (Fig. 1a).

### Exosomes promote angiogenesis in vitro

To assess the proangiogenic activities of exosomes released from BMSCs, we performed cell proliferation assays. Compared to those in the control group, MSC-Exos and DMOG-MSC-Exos significantly increased endothelial cell proliferation at days 1, 2, 3, and 4 (*P* < 0.05; Fig. 1b), with no significant difference between the two types of exosomes (*P* > 0.05). Next, we performed scratch wound healing assays to evaluate cell migration (Fig. 1c, d). After incubation for 6 h, HUVECs co-cultured with both DMOG-MSC-Exos and MSC-Exos migrated faster than the control group, and DMOG-MSC-Exos migrated noticeably faster than those in the other two groups (*P* < 0.05). After incubation for 12 h, scratches were completely covered by HUVECs in the DMOG-MSC-Exos group (all *P* < 0.05). Finally, to assess the proangiogenic potential of exosomes, tube formation assays were performed. HUVECs cultured with DMOG-MSC-Exos generated more cord-like structures on Matrigel than those cultured with MSC-Exos or PBS alone (Fig. 1e, f). These results indicate that the exosomes released from BMSCs stimulated by DMOG-enhanced angiogenesis in vitro but did not promote the proliferation of HUVECs.

### Exosome transplantation improves bone regeneration in critical-sized rat calvarial defects in vivo

**Fig. 2:**
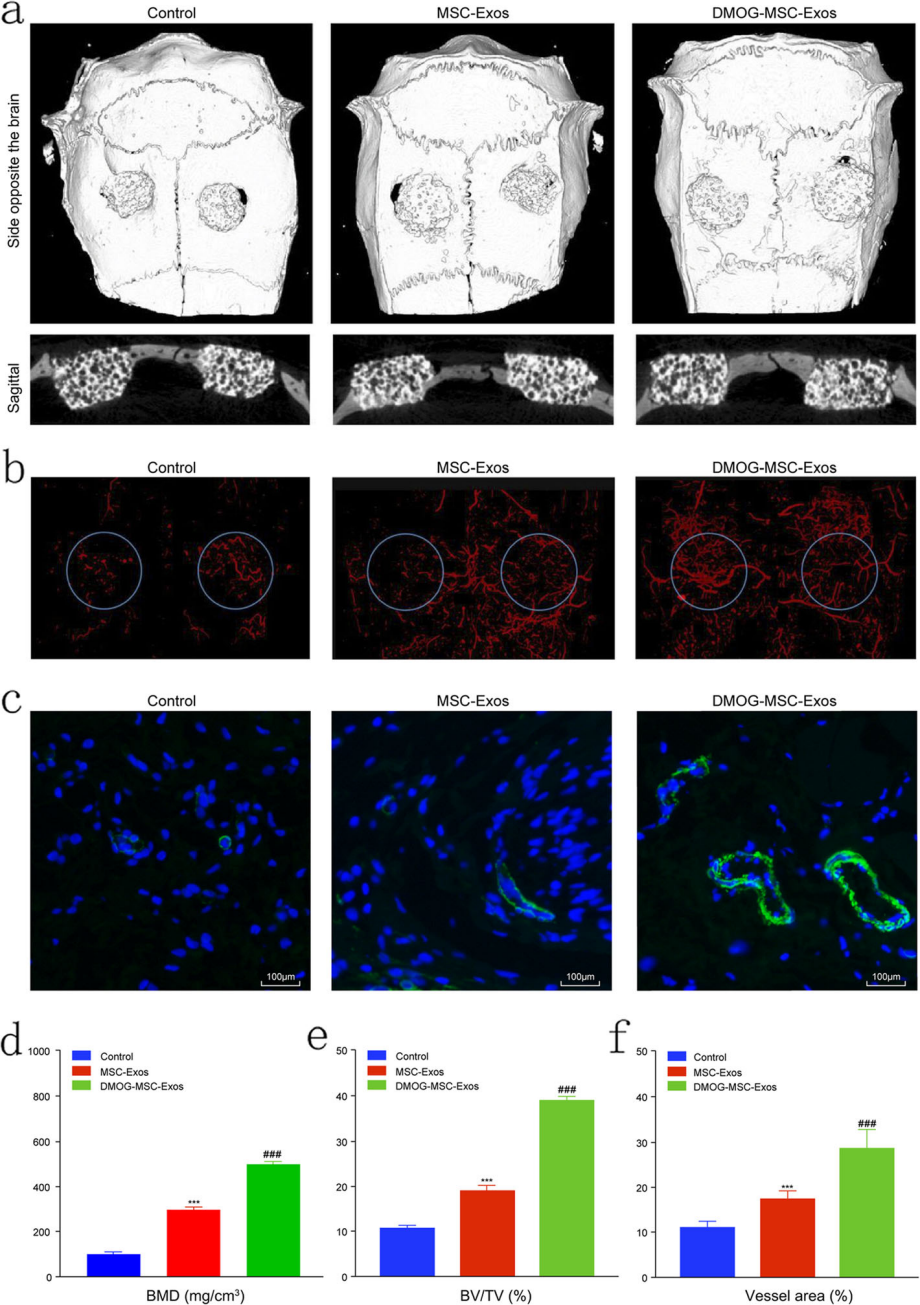
Micro-CT evaluation of repaired craniums and blood vessel formation at 8 weeks post-implantation. **a** Three-dimensional reconstruction and sagittal images showed different reparative effects of HA, MSC-Exos, and DMOG-MSC-Exos. **b** New blood vessels in calvarial defects are shown in three-dimensional reconstruction images. **c** CD31 immunohistochemistry of bone defect regions of all groups at 8 weeks post-surgery (green fluorescence indicates newly formed blood vessels). **d** Bone mineral density (BMD) of each group. **e** Bone volume-to-total volume ratio (BV/TV) in each group. **f** Morphometric analysis to determine local vessel area in bone defects (*P < 0.05, versus control; #*P* < 0.05, versus MSC-Exos)

In a critical-sized calvarial defect rat model, the morphology of newly formed bone was reconstructed by micro-CT. In the sagittal view, more newly formed bone filling the HA pores was observed in the DMOG-MSC-Exos group than in the MSC-Exos and HA groups (Fig. 2a). According to quantitative analysis of the newly formed bone, the BV/TV ratio and BMD in the DMOG-MSC-Exos group was markedly higher than that in the other two groups (Fig. 2e, d), indicating that the presence of exosomes released from BMSCs stimulated by DMOG improved the bone healing capacity in vivo.

### Neovascularization of defects

New vessel formation in the calvarial defects was visualized by Microfil perfusion. Three-dimensional micro-CT images showed that neovascularization in the DMOG-MSC-Exos and MSC-Exos groups was markedly greater than that in the HA group. Additionally, DMOG-MSC-Exos resulted in greater neovascularization than MSC-Exos (Fig. 2b). Quantification of the blood vessel areas confirmed these results (Fig. 2f). Additionally, newly formed vessels at the defect area were detected by CD31 labeling. The results show that both MSC-Exos and DMOG-MSC-Exos groups exhibited higher levels of angiogenesis than the control group. Furthermore, the detected microvessels were significantly larger in the DMOG-MSC-Exos group than in the MSC-Exos group (Fig. 2c).

### Fluorochrome labeling and histology/histomorphometry

**Fig. 3:**
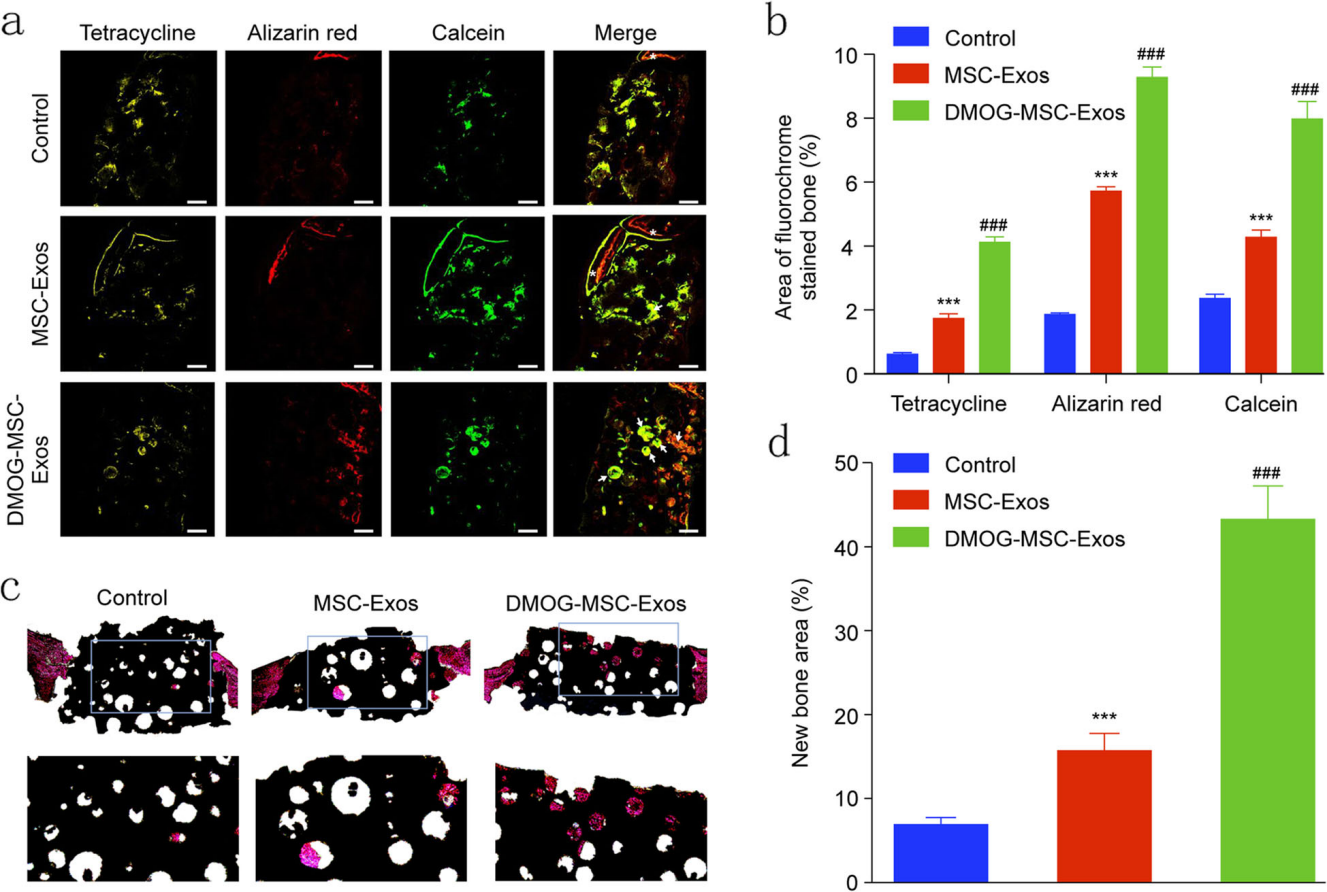
Histomorphometric analysis of new bone regeneration via fluorochrome labeling and histological analysis. **a** Staining with tetracycline (column 1, yellow) after 2 weeks, alizarin red (column 2, red) after 4 weeks, and calcein (column 3, green) after 6 weeks. Column 4 shows the merged images of the three fluorochromes for the same groups (scale bar = 100 μm, white arrows indicate round shapes, high intensity, and large mass of newly formed bone in HA pores; white asterisks indicate edges of bone defect). **b** Percentage of each fluorochrome area in each group. **c** Undecalcified craniums were sliced, and sections were stained with van Gieson’s picrofuchsin. New bone and HA are shown in red and black, respectively. **d** New bone area in each group (*P < 0.05, versus control; #*P* < 0.05, versus MSC-Exos)

New bone formation and mineralization were quantitatively analyzed at weeks 2, 4, and 6 post-surgery by fluorescence labeling (Fig. 3a, b). After 2 weeks, the percentage of tetracycline labeling in the DMOG-MSC-Exos group was greater than that in the other two groups. After 4 weeks, the percentage of alizarin red labeling was higher in the DMOG-MSC-Exos group than in the other two groups. Similarly, after 6 weeks, the percentage of calcein labeling in the DMOG-MSC-Exos group was significantly higher than that in the other two groups, with increased intensity, amount, and mass of newly formed round shape bone islands in HA pores. van Gieson’s picrofuchsin staining of the undecalcified craniums revealed that bone regeneration was markedly increased following the addition of DMOG-MSC-Exos (Fig. 3c, d). These results further suggest that the DMOG-MSC-Exos improved the bone healing capacity in vivo.

### Downregulation of PTEN plays a key role in migration and invasion of HUVECs

**Fig. 4:**
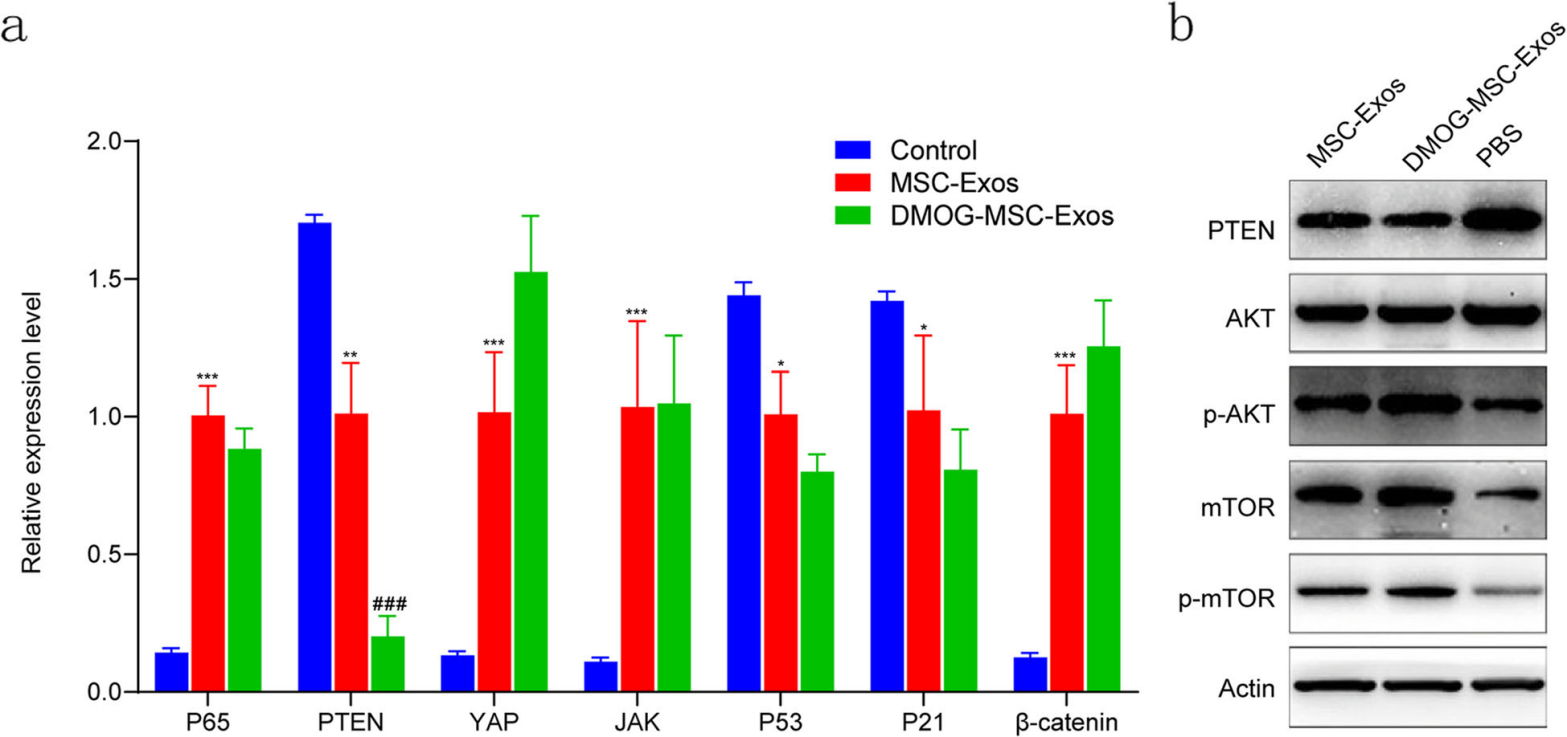
Mechanism of proangiogenic effects of DMOG-MSC-Exos. **a** HUVECs were treated with MSC-Exos with or without DMOG, treated with PBS as the control, mRNA levels of p53, p21, p65, Hippo-YAP, JAK-STAT, β-catenin, and PTEN were evaluated by qRT-PCR. **b** HUVECs were treated with MSC-Exos, DMOG-MSC-Exos, and PBS as a control; protein levels of members of the AKT/mTOR pathway were detected by western blotting. Actin was used as a loading control (*P < 0.05, versus control; #*P* < 0.05, versus MSC-Exos)

**Fig. 5:**
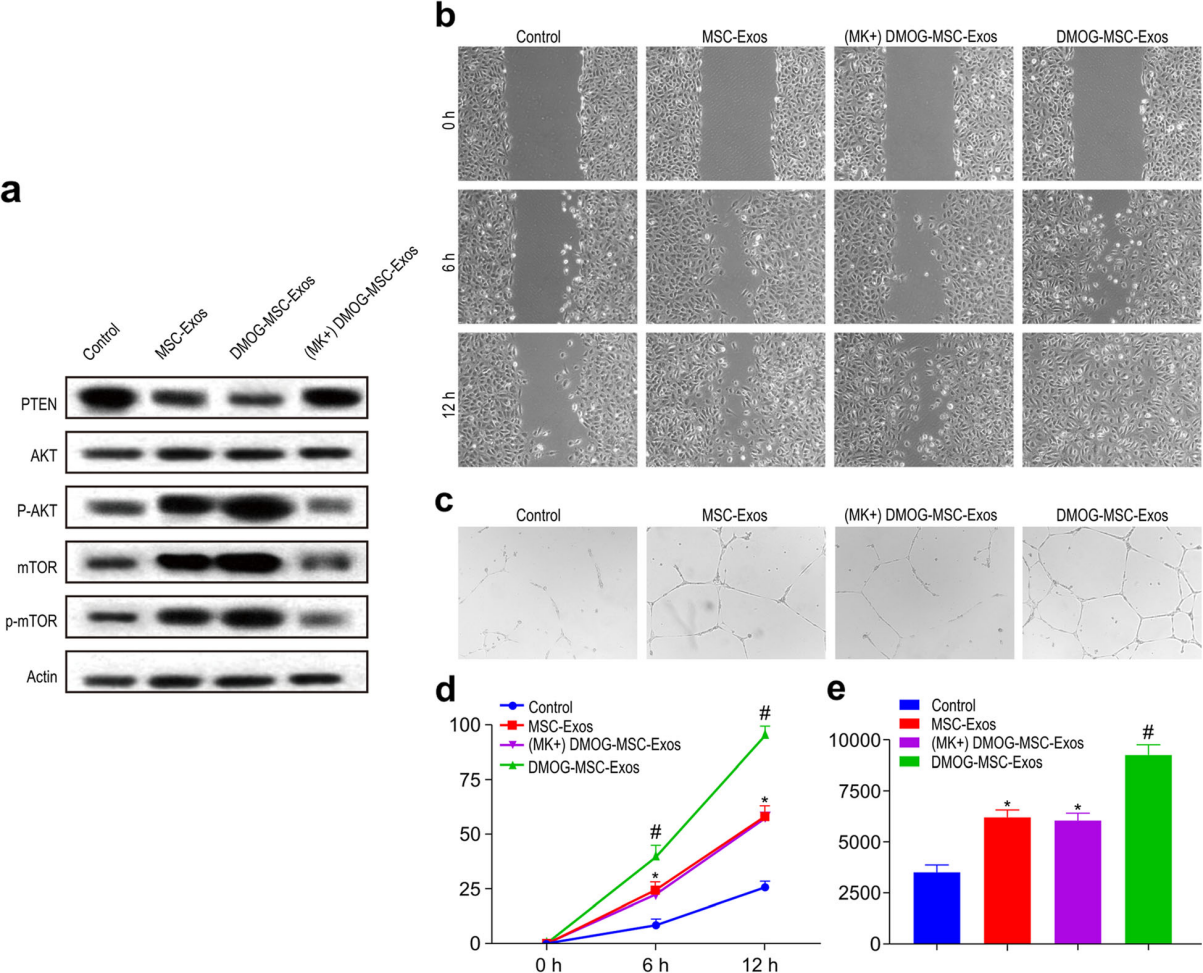
Proangiogenic effects of DMOG-MSC-Exos in HUVECS after AKT/mTOR pathway was blocked. **a** DMOG-MSC-Exos were treated with MK2206 and blocking of AKT/mTOR signaling was detected by western blotting. **b** Wound healing assay in HUVECs treated with exosomes (50 μg/mL). **c** Tube-forming capacity of HUVECs treated with exosomes (50 μg/mL). **d** Quantitative analysis of wound closure. **e** Quantitative analysis of total tube length (*P < 0.05, versus control; #*P* < 0.05, versus MSC-Exos)

The above results suggest that DMOG-MSC-Exos have superior proangiogenic properties to MSC-Exos and promote angiogenesis in HUVECs. To reveal the underlying mechanisms mediating this phenomenon, we assessed changes in the gene expression of several signaling molecules that regulate proliferation and migration, including p53, p21, p65, Hippo-YAP, JAK-STAT, β-catenin, and PTEN, in HUVECs following treatment with MSC-Exos or DMOG-MSC-Exos. Compared to the control group, both MSC-Exos and DMOG-MSC-Exos groups can significantly change the expression of these molecules critical for proliferation, differentiation, angiogenesis, and apoptosis. However, among these genes, the expression of PTEN exhibited the most significant change in the DMOG-MSC-Exos group than in the MSC-Exos group (Fig. 4a). *PTEN* is a well-defined tumor suppressor gene. It has also been reported that deficiency of PTEN contributes to the migration and invasion of HUVECs, leading to neovascularization [33]. To determine whether PTEN induced functional changes in DMOG-MSC-Exo-treated HUVECs, we investigated the downstream target of PTEN, the AKT/mTOR pathway (Fig. 4b), and we observed that DMOG-MSC-Exos markedly increased the expression levels of p-AKT, mTOR, and p-mTOR in HUVECs. To further elucidate the role of the AKT/mTOR pathway in this process, we additionally evaluated scratch wound healing and tube formation abilities of DMOG-MSC-Exos after the AKT/mTOR pathway was blocked with the Akt kinase inhibitor MK2206 (Fig. 5a). We found that the superior proangiogenesis ability of DMOG-MSC-Exos was absent, and the DMOG-MSC-Exos (MK+) group demonstrated neither better scratch wound healing nor better tube formation abilities than the MSC-Exos group (Fig. 5b, c).

## Discussion

In the present study, we determined whether exosomes derived from BMSCs preconditioned by a low dose of DMOG exert superior proangiogenic activity in bone regeneration and explored the underlying mechanisms involved. Our findings demonstrated that DMOG-MSC-Exos activated the AKT/mTOR pathway to stimulate angiogenesis in HUVECs, thereby contributing to bone regeneration and angiogenesis in a critical-sized calvarial defect rat model in vivo. These results suggest that stimulation with a low concentration of DMOG is a promising pretreatment for exosome-based approaches for critical-sized bone defects.

Previous studies have demonstrated several advantages of using exosomes derived from MSCs rather than MSCs in angiogenesis. Specifically, the use of exosomes reduces potential risks associated with cellular therapies, including ectopic tissue formation and immune rejection response. In this study, we established a critical-sized calvarial defect model in rats that is simple and reliable. Our data showed that the local application of DMOG-MSC-Exos improved bone regeneration and increased blood perfusion and the number of microvessels.

Several studies have revealed that exposure to an oxidative stress microenvironment induces the expression of proangiogenic proteins, not only in stem cells but also in their exosomes. For example, Atienzar-Aroca et al. [35] demonstrated that the levels of certain proteins and mRNAs in exosomes, such as vascular endothelial growth factor-1 and factor-2, were increased when RPE cells were exposed to oxidative stress; furthermore, when these exosomes interacted with endothelial cells, their angiogenic abilities were enhanced. In this study, we demonstrated that DMOG-MSC-Exos exhibited superior proangiogenic properties in vitro. However, interestingly, whereas DMOG-MSC-Exos promoted tube formation, they did not enhance cell proliferation.

Based on our results, the superior proangiogenic ability of exosomes conferred by preconditioning with DMOG may be mainly attributable to improvement in quality and activity of molecules in their cargo, not to increased number of released exosomes, because there was no significant differences between the DMOG-MSC-Exos and MSC-Exos groups in intensity of surface marker expression, size, shape, and number per milliliters of released exosomes. EVs, MVs, and apoptotic bodies also have the ability to package active cargo and deliver it to other cells, and their presence may interfere with the study of exosomes. However, the size of MVs and apoptotic bodies typically range from 100 to 1000 nm, and 50 to 5000 nm, respectively, in diameter, which are different from the qNano results obtained in this study [2, 3]. Based on surface marker expression and particle diameter results, which coincide with the described features of exosomes, the possible interference from MVs and apoptotic bodies may be very limited. Zhang et al. [14] demonstrated that hypoxia treatment of umbilical cord-derived MSCs can enhance their microvesicle production, which is different from what we find for exosomes in this study. One explanation for such phenomena may be the different origins of exosomes and microvesicles. Exosomal vesicles are formed by inward budding of the limiting membrane of early endosomes, which mature into multivesicular bodies (MVBs). MVBs are eventually either degraded by lysosomes or fused with the plasma membrane to release its contents into the extracellular space, including exosomes [8]. Whereas microvesicles are formed by direct outward budding, or pinching, of the plasma membrane, and it has been verified that microvesicle production is sensitive to the physiological state and microenvironment of the source cell, no such sensitivity is observed in exosome production [15, 29].

To better assess the proangiogenic potential of DMOG stimulation, DMOG-MSC-Exos were used to treat critical-sized bone defects and showed better results than MSC-Exos. To investigate the underlying mechanisms mediating this phenomenon, we assessed the expression of several signaling molecules in HUVECs treated with DMOG-MSC-Exos. To identify pathways that play key roles in the DMOG-MSC-Exos-induced enhancement of the angiogenic ability of HUVECs, we evaluated the activity of many critical molecules of several classical pathways that regulate cell proliferation, differentiation, and apoptosis. Compared to the control group, MSC-Exos significantly elevated the expression of key components of classical pathways related to proliferation and osteogenic and angiogenic differentiations, including YAP of the Hippo pathway, JAK of the JAK/STAT pathway, p65 of the NF-κB pathway, and β-catenin of the wnt pathway. In contrast, PTEN and key factors that regulate apoptosis such as P53 and P21 were significantly downregulated, which is consistent with previous studies of MSC- and MSC-derived exosomes [4, 5, 12]. However, in our study, no obvious differences were seen between MSC-Exos and DMOG-MSC-Exos groups, except for PTEN, which exhibited the most significant downregulation in the DMOG-MSC-Exos group. PTEN is an endogenous negative regulator of the PI 3 kinase signal pathway, inducing the inhibition of Akt kinase and mTOR kinase and thus inhibiting angioproliferation of endothelial cells [36, 37]. On the other hand, when the AKT/mTOR pathway is activated, mTOR activation can lead to the downregulation of PTEN, thus further alleviating its inhibition effect on AKT/mTOR pathway, which creates a positive feedback loop to facilitate the activation of the pathway [38]. Thus, based on to the qRT-PCR results, the AKT/mTOR pathway appears to be most involved in this angiogenic enhancement ability of DMOG-MSC-Exos. Western blotting analysis shows, as expected, that the expression of p-AKT, mTOR, and p-mTOR were significantly higher in the DMOG-MSC-Exos group. To further evaluate the possibly important role of the AKT/mTOR pathway in this process, in vitro angiogenesis evaluation was repeated after the AKT/mTOR pathway was blocked. Our results show that DMOG-MSC-Exos no longer demonstrated a superior angiogenesis enhancement effect than MSC-Exos when the AKT/mTOR pathway was blocked. We therefore conclude that DMOG-stimulated BMSC-derived exosomes can enhance angiogenesis and osteogenesis mainly by targeting the AKT/mTOR pathway. However, the detailed mechanisms require further elucidation, and this represents a limitation of this study. Additionally, it is unclear whether the levels of certain exosome cargo, such as miRNAs or proteins, are increased by DMOG pretreatment and contribute to the proangiogenic potential of DMOG-MSC-Exos.

## Conclusions

In conclusion, our study demonstrated that DMOG-MSC-Exos promote neovascularization via the AKT/mTOR pathway and enhance bone regeneration in critical-sized bone defects in rats. Thus, low doses of DMOG may trigger exosomes to exert enhanced proangiogenic activity in cell-free therapeutic applications. Importantly, the optimum dose of DMOG should be investigated using concentration gradient experiments in future studies. Furthermore, the detailed mechanisms of its ability to enhance angiogenesis and osteogenesis also need further elucidation.

## Supplementary information

**Additional file 1:** Characterization of exosomes. Figure S1 (a) CD9, CD63, TSG101, and GM130 expression in exosomes was detected by western blotting. (b) TEM photomicrographs of exosomes. Scale bar = 100 nm. (c) Estimated sizes of exosomes. (TIF 1365 kb)

## Data Availability

The datasets used and/or analyzed during the current study are available from the corresponding author on reasonable request.

## Abbreviations

BMSCs: Bone marrow-derived MSCs
DMOG: Dimethyloxaloylglycine
EVs: Extracellular vesicles
HIF: Hypoxia-inducible factor
HUVECs: Human umbilical vein endothelial cells
MSC: Mesenchymal stem cell
MVBs: Multivesicular bodies
MVs: Microvesicles
OD: Optical density
