# Supplementary figures and images for: Dimethyloxaloylglycine-stimulated human bone marrow mesenchymal stem cell-derived exosomes enhance bone regeneration through angiogenesis by targeting the AKT/mTOR pathway
Source: Stem Cell Res Ther. 2019 Nov 20;10:335. doi: 10.1186/s13287-019-1410-y (PMC6869275; doi:10.1186/s13287-019-1410-y)

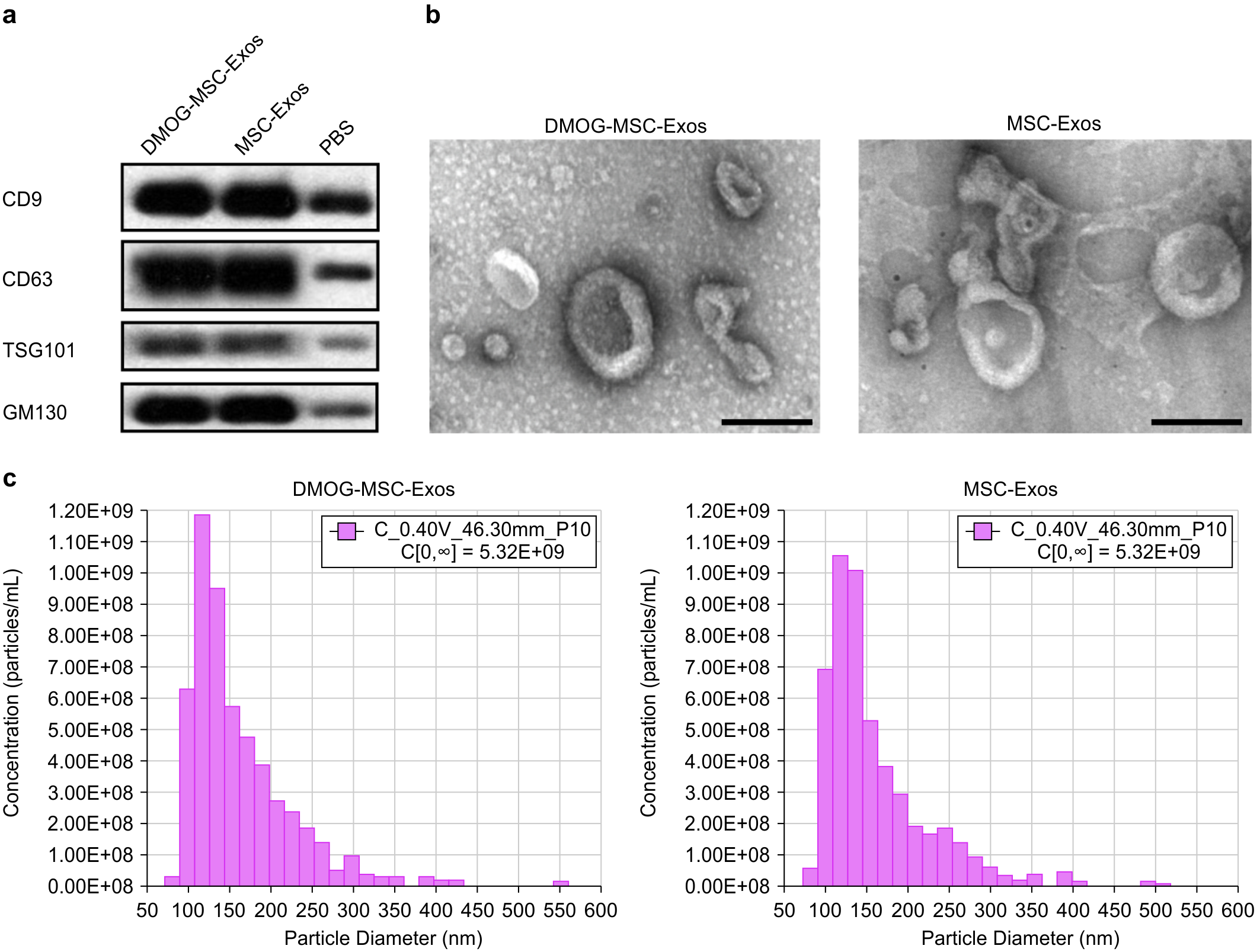

Supplement: Supplementary file 1 — Additional file 1: Characterization of exosomes. Figure S1 (a) CD9, CD63, TSG101, and GM130 expression in exosomes was detected by western blotting. (b) TEM photomicrographs of exosomes. Scale bar = 100 nm. (c) Estimated sizes of exosomes. (TIF 1365 kb) [file 13287_2019_1410_MOESM1_ESM.tif]
